# Effect of traditional Chinese medicine formula Guilu Xian on in vitro fertilization and embryo transfer outcome in older women with low prognosis: study protocol for a prospective, multicenter, randomized double-blind study

**DOI:** 10.1186/s13063-021-05867-5

**Published:** 2021-12-13

**Authors:** Yingjie Ma, Xianling Cao, Jingyan Song, Dandan Gao, Xinlei Wang, Li Li, Zhengao Sun

**Affiliations:** 1 First College of Clinical Medicine, Shandong University of Traditional Chinese Medicine, Jinan, China; 2 College of Traditional Chinese Medicine, Affiliated Hospital of Shandong University of Traditional Chinese Medicine, Jinan, Shandong China; 3 Fudan University, Shanghai, China; 4 Reproductive Medicine Center of Integration of Traditional and Western Medicine, Affiliated Hospital of Shandong University of Traditional Chinese Medicine, No. 42, Wen Hua Xi Road, Lixia District, Jinan, 250011 China

## Abstract

**Introduction:**

In recent years, the prevalence of infertility has significantly increased and has become a global reproductive health problem. The female ovarian reserves have been shown to decrease progressively with an increase in age. Besides, the rate of embryo implantation and clinical pregnancy also decreases. Traditional Chinese medicine has been widely applied in assisted reproductive technology. It is reported to have a significant influence on improving the quality of oocytes, improving endometrial receptivity, increasing clinical pregnancy rate, reducing pregnancy-related complications, etc. Therefore, this study will investigate the effect of Guilu Xian, a traditional Chinese medicine formula on IVF-ET outcome in older women with low prognosis.

**Methods and analysis:**

This trial is a prospective, multicenter, randomized double-blind clinical trial. A total of 120 infertile patients with low prognosis and receiving IVF or ICSI in 3 public hospitals in China will be randomly divided into two parallel groups: Guilu Xian group (*n* = 60) and placebo group (*n* = 60). Patients in both groups will be treated with antagonist regimens to promote ovulation, and all the patients will be required to take the medication from the 2nd to 4th day of the menstrual cycle to the day of egg retrieval. A comparison of the total number of oocytes obtained, the fertilization rate, clinical pregnancy rate, embryo quality, embryo implantation rate, and early spontaneous abortion rate between the experimental group and the placebo group will be performed.

**Trial registration:**

Chinese Clinical Trials Registry ChiCTR1900028255. Registered on 16 December 2019.

**Supplementary Information:**

The online version contains supplementary material available at 10.1186/s13063-021-05867-5.

## Introduction

Infertility affects about 8 to 12% of couples of childbearing age globally, and this accounts for more than 186 million people, and the majority of them being residents of developing countries. Increasing age in women is one of the most powerful negative predictors of fertility [1]. Rapid social and economic development has led to a gradual increase in the number of people with late marriages and childbearing, especially with the liberalization of the policy of “comprehensive second child.” This has increased in the number of older women seeking assisted reproductive technology (ART) treatment. The reproductive ability of women gradually decreases with increasing age [2]. Irreversible aging of the ovaries may lead to diminished ovarian reserves (DOR), however, in vitro fertilization-embryo transfer (IVF-ET) technology has become an effective treatment for DOR in older women. Although IVF-ET is increasingly being used to deal with infertility, the clinical pregnancy and live birth rate among older women with a poor ovarian response to exogenous gonadotropin stimulation (DOR) remains unsatisfactory.

Low prognosis is considered one of the most common and intractable problems of IVF-ET treatment occurring in about 47% of women. Out of these, 55% are women above the age of 35 years and with insufficient ovarian reserves [3–5]. The main cause of diminished ovarian response declines in the number of follicles responsive to FSH. This phenomenon is most common in older women with severely decreased ovarian reserves due to accelerated follicle loss [6]. However, in some cases, patients with good ovarian reserves may experience poor ovarian response owing to the gonadotropin dosage used [7] or due to the impact of genetic polymorphisms of endogenous gonadotropins and their receptors [8–10]. These factors change the response of recruitable follicles to exogenous gonadotropins, and this is associated with several problems such as a small number of eggs, poor embryo quality, poor pregnancy outcomes, etc. Therefore, patients with low prognosis are in need of high gonadotropin (Gn) dose and cycles for ovarian stimulation during the IVF cycle. This causes emotional and physical pain and financial burden to couples [6]. Currently, there are no effective ways of improving clinical pregnancy and live birth rates for patients with increased age and poor ovarian reserve [11]. The follicular fluid provides an important microenvironment in the growth and development of oocytes. Several metabolites in the follicular fluid are associated with the oocyte’s ability to undergo fertilization, embryonic development, and pregnancy outcome [12].

Traditional Chinese medicine has a significant effect on improving the quality of oocytes, improving the endometrial receptivity, increasing the clinical pregnancy rate, and reducing pregnancy-related complications. Tortoise deer erxian ointment, also known as tortoise deer erxian gum, was first recorded by Wang Sancai, a Chinese physician in the Ming Dynasty(1569). Guilu Xian reported the addition of turtle shell glue to the original “Guilu Erxian Ointment” and adjusted the dosage to strengthen the effect of nourishing yin and latent yang. The prescription is based on the tortoise shell glue and turtle shell glue as the main medicine, which functions in tonifying the kidney and as a nourishing essence and yin and latent yang. The application of deer horn glue is based on the fact that “those who are good at tonifying yin must seek yin in yang, then yin is born in yang, and the source of spring is not thirsty” [13]. This maximizes the tonifying effect in the balance of yin and yang. Therefore, Guilu Xian has the effect of tonifying the kidney essence, nourishing yin and latent yang, replenishing qi and nourishing spirit, and delaying aging. According to TCM theory, the pathogenesis of older women with low prognosis is deficient kidney essence, spleen qi deficiency, liver depression, and blood deficiency. “Tonifying the kidney, regulating the liver and invigorating the spleen” is the main treatment method, which corresponds to the tortoise deer fairy syndrome previously described. Recent studies have shown that TCM formula Guilu Erxian ointment has a good effect on the treatment of the reproductive system, and has an estrogenic effect [14–17]. However, there is no clear case on how to improve prognosis among older women. Therefore, there is necessary that a well-designed randomized controlled trial to confirm the effectiveness and safety of Guilu Xian in improving IVF-ET outcome in older women with low prognosis.

## Methods and analysis

### Aims of the study

To explore the effect of Guilu Xian, a traditional Chinese medicine formula on the IVF-ET outcome in older women with low prognosis.

### Trial design

This is a prospective, randomized, placebo-controlled, double-blind, superiority trial assigned in a 1:1 ratio. The study institutions were the Genetic and Reproductive Center of Traditional Chinese and Western Medicine, Affiliated Hospital of Shandong University of Traditional Chinese Medicine, the Second Affiliated Hospital of Shandong University of Traditional Chinese Medicine, and Jinan Central Hospital. The project leader regularly coordinates and communicates with each unit to understand the progress of the project and ensure the smooth progress of the project and the guarantee of quality. In order to ensure that there are enough participants, we will take measures such as providing free basic examinations, free drugs, and car allowance to better attract participants to join.

The inclusion criteria for this study will be patients aged between 35 and 42 years old, with insufficient ovarian reserve parameters (The POSEIDON criteria AFC < 5, AMH < 1.1 ng/mL) [18], and poor ovarian response (that is, the number of oocytes obtained after standard ovarian stimulation is less than 3). Patients should meet the diagnostic criteria of western medicine infertility, that is, women living without contraception for at least 12 months who are not pregnant are infertile [19].

The exclusion criteria are women with BMI ≥ 25 kg/m^2^; women who suffer from genetic diseases that are not suitable for childbearing as stipulated in the Maternal and Child Health Care Law; women with severe endometriosis, adenomyosis, and immune infertility; with untreated hydrosalpinx; with congenital or acquired abnormal development of the uterus and severe deformities of other reproductive organs; women with a history of endocrine dysfunction such as reproductive system tumor, thyroid dysfunction, and hyperprolactinemia; women receiving ovarian stimulation therapy or OCP in recent 3 months; women with a previous history of gynecological surgery such as the ovary; and all other contraindications of assisted reproductive technology.

The discontinuation criteria will be women who have adverse reactions such as nausea and obesity occurring during medication, women with no dominant follicular growth, early follicular ovulation, and no egg acquisition during ovarian stimulation. Three days after egg harvesting, the embryo was not formed.

### Technical route

See Annex 1 for details.

### Schedule of the study process

See Annex 2 for details.

### Case registration and allocation

The clinicians being involved in the study will record the basic AFC and AMH values of the patients after obtaining informed consent from the participants. Upon meeting the inclusion criteria, the patients will be randomly distributed in groups with the order of registration, and the clinicians are responsible for recruiting patients in sequence and will use case questionnaires to record their identity, age, and other basic information.

The clinicians will give medication to participants within 3 days after screening. Data will be recorded on drug distribution and use, and timely reports provided to the clinicians on the drug dose and patients’ discomfort during the process.

This study will include 120 patients who will be recruited and randomly divided into two groups using the R language version 3.5.1: the elderly test group and the elderly control group. Sixty, patients will be treated with Guilu Xian and 60 with the placebo. The elderly and the experimental group will be treated with an antagonist, and the tortoise deer fairy and placebo will be administered from the third day of menstruation before IVF.

### Ovarian stimulation and egg extraction and transplantation

The flexible GnRH antagonist regimen will be utilized in the COS regimen in both groups. On day 3 of the cycle, gonadotropin (Serono) or urinary gonadotropin (Lubao biochemical Pharmaceutical Company, Zhuhai Lizhu Group) 300 IU will be administered, and regular monitoring of follicular development performed. When ≥ 1 dominant follicle is at least ≥ 14 mm in diameter or LH 10 U/L, GnRH-ant will be administered. However, when ≥ 2 dominant follicles are ≥ 18 mm in diameter, urogenic hCG 8000~10,000 U or r-hCG 250 μg will be administered. Transvaginal ultrasound-guided paracentesis will be performed 36 h after the injection of HCG. Fresh or frozen embryo transfer will be based on the patient’s characteristics such as intima growth value, *P* value, and embryo score. Following transplantation, progesterone will be injected into the supporting luteal muscle at 40 mg/ day or application of progesterone vaginal sustained-release gel at 90 mg/day. Drug administration will be stopped from the day of transplantation to the 10th week of gestation.

### Estimation of the sample size

In a recent study in China, it was reported that in conventional IVF or ICSI, the total number of retrieved eggs in the treatment group (259.77±25.45) was significantly higher than that in the placebo group (235.59±26.44). In this study, the number of eggs obtained in the Guiluxian group was 11 under the original hypothesis and 9 under the alternative hypothesis (9 in the placebo group). The bilateral *Z* test of the combined variance will be used to estimate the sample size at 0.05 level of significance. The ratio between groups will be1:1. The minimum sample size found for each group is 49 people, hence a total of 98 people. Besides, 90% of the detection ability will be obtained, and the difference in ratio between groups is 0.15. Considering a dropout rate of 15%, we will be expected to enroll 120 participants, with 60 participants in each group (see supplementary files 1 and 2 for details).

### Randomization and blinding

After signing the informed consent form, volunteers participating in the trial were randomly assigned to one of the two trial groups in a 1:1 ratio using stratified zone group randomization, and random numbers were generated using R software version 3.5.1. A strictly confidential list of randomization numbers will be maintained by the dosing center staff. Randomization will be stratified by trial center in different regions. The active drug (Guilu Xian) and placebo will be manufactured with similar appearance and smell. The clinicians and patients will be blinded to the drug allocation until the end of the study. If there are multiple adverse reactions in the trial, we will take emergency unblinding measures to find the cause of adverse events. To achieve high precision, the trial will follow the Consolidated Standards of Reporting Trials (CONSORT) 2010 statement [20] and the updated guidelines for reporting parallel-group randomized trials of the traditional Chinese medicine CONSORT [21].

### Intervention

The Guilu Xian/placebo will be given to the patients at 5 g three times daily from the third day of their menstruation period before IVF for 40 days (provided by the preparation room of Affiliated Hospital of Shandong University of Traditional Chinese Medicine, and the extraction procedure will be based on the standard of preparation room SOP). Drug composition: tortoise glue, turtle glue, antler glue, Chinese wolfberry, American ginseng, Cornus officinalis, Hawthorn seed, jujube.

### Concomitant care

All subjects will not be allowed to take any other Chinese herbal supplements or nutritional supplements that may increase ovarian response for 3 months prior to the start of the study. Such drugs or nutritional supplements may affect the therapeutic effect of modified Guilu Erxian ointment.

### Placebo

The placebo was produced by the Affiliated Hospital of Shandong University of Traditional Chinese Medicine. The placebo was not different from Guilu Xian in appearance, color, and odor and had no active ingredients. On the one hand, can prevent participants from recognizing being assigned to a placebo group, and this may reduce participants’ compliance and on the other hand, it can prevent the potential beneficial effects of using vitamins as a placebo.

### Patient compliance

Assessment of patient’s compliance, including the bottle count of the drugs and weekly telephone follow-up, will be performed to find out if the participants are taking the medicine as agreed and any reasons for non-compliance. All unused drugs will be counted and registered. We will exclude patients with less than 70% compliance (i.e., in 40 days of treatment with Guilu fairy, while actually taking it for less than 28 days). They will not be included in the final analysis, but will be included in the intention-to-treat analysis.

### Patient and public involvement

Patients and public were not involved in developing the research questions nor the study design. Moreover, they will not participate in the recruitment exercise or the conduct of the study. Results of the study will be disseminated to participants and their families via telephone and patient organization platforms.

### Outcome measurement

The primary outcome will be the number of eggs obtained, and the secondary outcome will include the 2PN number, cleavage number, grade I embryo number, implantation rate, abortion rate, persistent pregnancy rate, trigger day ≥ 14 mm follicles, Gn days, and Gn.

### Data analysis

All data are reported as mean ± standard deviation (SD). Patients with low adherence will not be included in the final analysis, but will be included in the intention-to-treat analysis. Two sets of analyses will also be performed for non-adherence and missing outcomes. The full analysis set (FAS) will include all patients who are randomized and will proceed according to the intention-to-treat principle. The protocol-compliant set will include all patients in the FAS who completed follow-up and did not experience a serious protocol violation.

For baseline comparisons, differences in means of continuous data between the placebo and intervention groups will be evaluated using independent Student’s *t* tests or Mann-Whitney *U* tests, depending on whether the data are normally distributed, and frequencies will be compared using chi-square tests. For longitudinal data with baseline, we will evaluate mean differences between and within groups using repeated measures analysis of covariance (ANCOVA). All tests will be two-tailed and ratios between the two groups will be compared using Fisher’s exact rate method with a significance level defined as a value < 0.05.

### Data management

Specialized data management physicians in each reproductive center will collect information on the patient number and various data of the patients during the trial to ensure that the information will not be disclosed. All data collected will be saved for 5 years after publication. Electronic data will be stored in password-protected computers and access will only be allowed to the principal investigator. The data were uniformly managed by the database of the Reproductive Center of Shandong University of Traditional Chinese Medicine. Data obtained will only be restricted for use in this study.

### Supervision and management

We have also established a Data Monitoring Committee (DMC). The DMC is composed of the director of the Department of Reproductive Center of Shandong Provincial Hospital of Traditional Chinese Medicine as the chairman and the heads of the other two study sites as independent members. It is the responsibility of the DMC to monitor data, assess adverse events and cases of abnormal liver and kidney function results post-intervention on a regular monthly basis, review the core trial process, conduct interim analyses (the interim analysis includes the evaluation of the patient's follicular growth status, the number of retrieved oocytes and the incidence of adverse events. If the incidence of events of early follicular ovulation and absence of follicles after stimulation is as high as 30%, or the incidence of adverse events is as high as 10%, the trial will be terminated and the reasons for this will be analyzed.), and discuss relevant protocol amendments.

### Adverse events

The safety analysis will be performed on all participants who will have received at least one Guilu Xian treatment.

Regular quality assurance monitoring will be carried out for any adverse effects and to ensure that the study is safe and in line with the implementation plan. This will also ensure that the data are accurately recorded and stored. Any serious adverse effects will be reported to the principal investigator with immediate effect. We will compensate the participants accordingly according to the “Regulation on the Handling of Medical Malpractice” in China.

### Ethical approval and consent

This study has been approved by the Reproductive Medicine Ethics Committees of the Affiliated Hospital of Shandong University.

Clinicians will receive written informed consent from each patient participating in the study before the study commences. This study was approved by the Ethics Committee of the Reproductive and genetic Center of Integrated traditional Chinese and Western Medicine in Jinan City, Shandong Province, China.

## Discussion

Previous studies have confirmed that traditional Chinese medicine formula Guilu Erxian ointment is effective in the treatment of male impotence, semen abnormalities, female dysfunctional uterine bleeding, peri-menopausal osteoporosis, and other reproductive system diseases. A recent study found that Jiawei Guilu Erxian ointment effectively increased the number of elementary ovarian follicles in mice with primary ovarian insufficiency [22]. However, Guilu Xian treatment has not been reported in an older population of infertile women. Therefore, this prospective, multi-center, randomized double-blind clinical trial will investigate the effect of Guilu Xian, a traditional Chinese medicine formula on IVF-ET outcome in older women with low prognosis.

This study is a multi-center prospective clinical trial with certain limitations. The trial will not completely rule out clinical treatment bias. It will be difficult to guarantee that patients have similar backgrounds when comparing the two groups.

There is no current study to predict the impact of the Chinese Medicine Guiluxian on elderly infertile patients. Therefore, a prospective study is required to determine the feasibility and effectiveness of the turtle deer fairy.

### Protocol amendments

If major modifications are planned, such as inclusion criteria, exclusion criteria, and analysis of trial outcomes, we will notify the trial registration center and journal through the official website. For others such as investigators, trial participants, or regulators, we will notify them by telephone.

### Trial status

The trial was registered at the Chinese Clinical Trial Registry (ChiCTR) which is assigned to be the representative registry of China to join WHO ICTRP in 2007.

This trial is at version 1.3, 16 December 2019 (ChiTR).

The actual study start date was 1 July 2020 and the anticipated study end date is 31 December 2021. The recruitment start date was 15 October 2020; the anticipated recruitment end date is 31 January 2022.

### Data dissemination

The findings of this study will be widely disseminated through conference papers, research reports, and academic publications.

## Supplementary Information

**Additional file 1.** Technical route.

**Additional file 2.** Schedule of the study process
