# Supplementary figures and images for: Effect of traditional Chinese medicine formula Guilu Xian on in vitro fertilization and embryo transfer outcome in older women with low prognosis: study protocol for a prospective, multicenter, randomized double-blind study
Source: Trials. 2021 Dec 13;22:917. doi: 10.1186/s13063-021-05867-5 (PMC8667436; doi:10.1186/s13063-021-05867-5)

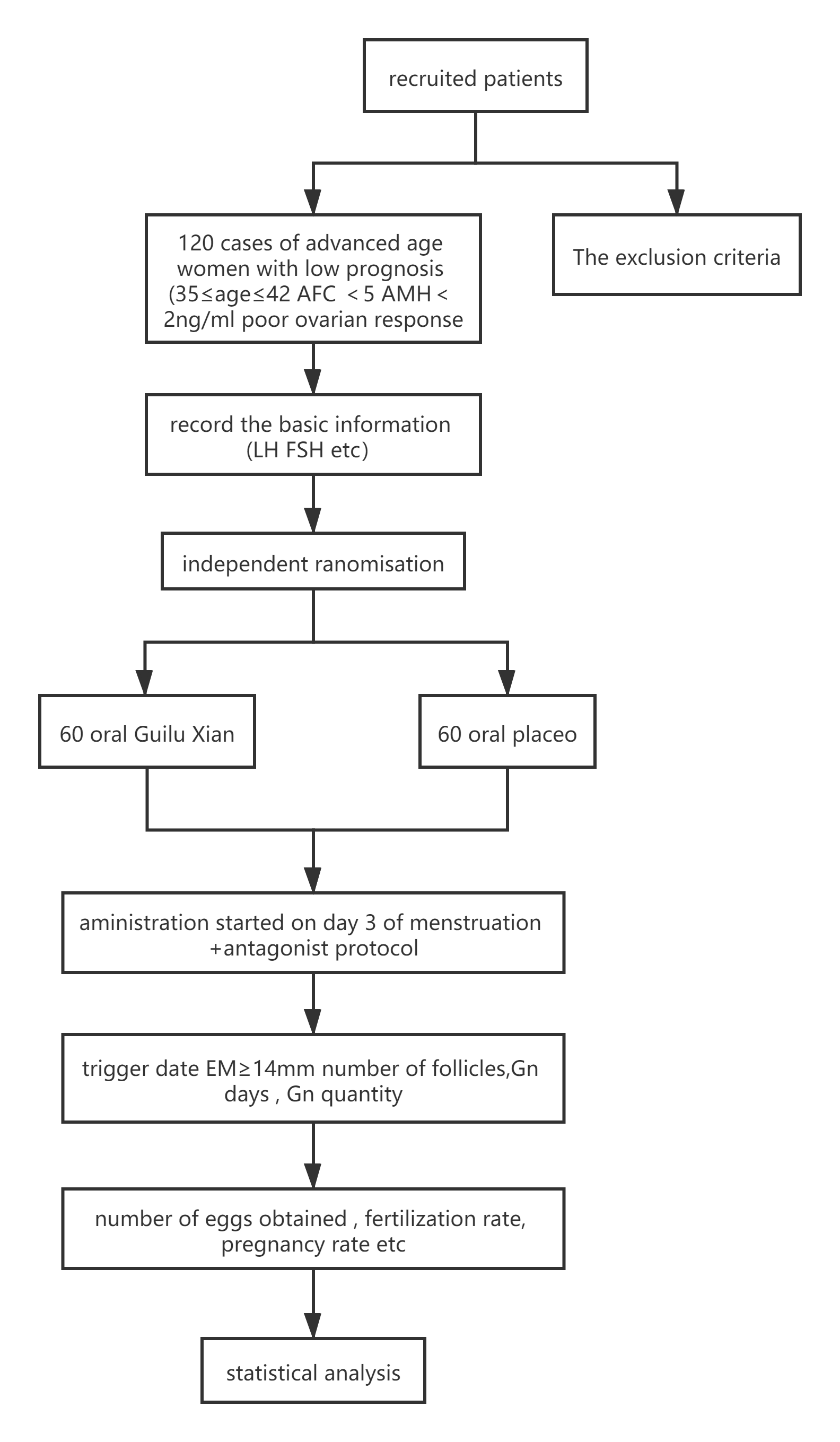

Supplement: Supplementary file 1 — Additional file 1. Technical route. [file 13063_2021_5867_MOESM1_ESM.docx]
