# Supplementary material for: Effect of traditional Chinese medicine formula Guilu Xian on in vitro fertilization and embryo transfer outcome in older women with low prognosis: study protocol for a prospective, multicenter, randomized double-blind study
Source: Trials. 2021 Dec 13;22:917. doi: 10.1186/s13063-021-05867-5 (PMC8667436; doi:10.1186/s13063-021-05867-5)
Supplement: Supplementary file 2 — Additional file 2. Schedule of the study process [file 13063_2021_5867_MOESM2_ESM.docx]

|  | **STUDY PERIOD** | | | | | | | |
| --- | --- | --- | --- | --- | --- | --- | --- | --- |
|  | **Enrolment** | **Allocation** | **Post-allocation** | | | | | **Close-out** |
| **TIMEPOINT**** |  | ***0 weeks*** | ***1-5 weeks*** | ***6-10 weeks*** | ***11-15 weeks*** | ***16-20 weeks*** | ***21-25 weeks*** |  |
| **ENROLMENT:** |  |  |  |  |  |  |  |  |
| **Eligibility screen** | ∨ |  |  |  |  |  |  |  |
| **Informed consent** | ∨ |  |  |  |  |  |  |  |
| **Basic information login** | ∨ |  |  |  |  |  |  |  |
| **Double-blind assignment** |  | ∨ |  |  |  |  |  |  |
| **INTERVENTIONS:** |  |  |  |  |  |  |  |  |
| **Guilu Xian treatment** |  |  |  |  |  |  |  |  |
| **Placebo treanment** |  |  |  |  |  |  |  |  |
| **ASSESSMENTS:** |  |  |  |  |  |  |  |  |
| **Basic information**  **(age,BMI,AFC,AMH, FSH,etc)** | ∨ |  |  |  |  |  |  |  |
| **Main indicators**  **(number of follicles)** |  |  |  | ∨ |  |  |  |  |
| **Secondary endpoints**  **(2PN number, implantation rate, persistent pregnancy rate,etc)** |  |  |  |  |  |  |  |  |
| **Fetal Development Assessment** |  |  |  |  |  |  |  | ∨ |
| **Maternal assessment of hepatic and renal function** |  |  |  |  |  |  |  | ∨ |
| **Storage of biological specimens for genetic or molecular analysis** |  |  |  |  |  |  |  |  |
